# Illuminating the genome-wide activity of genome editors for safe and effective therapeutics

**DOI:** 10.1186/s13059-018-1610-2

**Published:** 2018-12-22

**Authors:** Yong Cheng, Shengdar Q. Tsai

**Affiliations:** Department of Hematology, St. Jude Children’s Research Hospital, 262 Danny Thomas Place, Memphis, TN 38105 USA

## Abstract

Genome editing holds remarkable promise to transform human medicine as new therapies that can directly address the genetic causes of disease. However, concerns remain about possible undesired biological consequences of genome editors, particularly the introduction of unintended ‘off-target’ mutations. Here, we discuss both important considerations for therapeutic genome editing and our understanding of the functional impact of undesired off-target mutations. An important challenge for the future will be the development of new approaches for predicting and defining the probable function of unintended genome-editing mutations, which will inspire confidence in the next generation of promising genome-editing therapies.

## Introduction

Genome editing is a transformative, broadly applicable technology for making targeted DNA modifications in the genomes of living cells with promise to unlock fundamentally new treatments for human genetic diseases. For example, clinical trials have been initiated to test the safety of a genome-editing approach that has the potential to cure HIV by disruption of *CCR5,* a gene encoding a co-receptor for HIV. Other promising therapeutic genome-editing approaches include the engineering of better T cells for cancer immunotherapy [1] or the editing of human hematopoietic stem and progenitor cells (HSPCs) for the treatment of hemoglobinopathies such as sickle cell disease or beta-thalassemia [2].

Current genome editors function either by cutting the DNA itself (nucleases) or by inducing point mutations through the recruitment of natural or engineered deaminases (base editors). There are four major classes of genome-editing nucleases: meganucleases [3], zinc-finger nucleases (ZFNs) [4], transcription activator effector-like nucleases (TALENs) [5], and clustered regularly interspaced palindromic repeats (CRISPR)-Cas RNA-guided nucleases [6]. Nuclease-induced DNA double-strand breaks (DSBs) can be repaired by the endogenous cellular DNA repair machinery, which has a bias towards insertion/deletion (indel) mutations associated with error-prone non-homologous end-joining (NHEJ) over precise homology-directed repair. Base editors are engineered enzymes that are a fusion of a CRISPR-Cas enzyme (used for its DNA-binding properties), a deaminase and, if applicable, a uracil-glycosylase inhibitor. In general, all genome-editing enzymes function by inducing targeted DNA damage that can be converted into useful mutations by the cells own DNA damage repair pathways.

Like many biological enzymes, genome editors do not possess perfect specificity for their targets and as a result may introduce unintended ‘off-target’ mutations into the genome. Off-target mutagenesis has been observed for all classes of genome editors used to date: meganucleases, ZFNs, TALENs, CRISPR-Cas nucleases, and base editors. However, by careful design, deployment of strategies to improve specificity [7–14], and analysis of genome-wide activity (previously reviewed by Tsai and Joung [15]) in many cases it is possible to avoid introducing detectable levels of off-target mutagenesis.

## Safety considerations for therapeutic genome editing

It is important to see the big picture and fully understand the consequences of harnessing powerful genome-editing enzymes to modify the genomic DNA of living cells. Unintended adverse consequences of therapeutic genome editing could jeopardize warm public support for this entire class of promising new therapies. For example, inadvertent activation of proto-oncogenes could predispose patients to cancer, a possible adverse outcome similar to that observed with the use of ɣ-retroviral vectors used in gene therapy for several inherited immunodeficiencies [16]. Alternatively, delivery agents or even the genome editors themselves could induce a cellular or immune response. In this Opinion, we focus on the direct intended and unintended consequences of the catalytic activity of genome editors.

There is no ‘magic number’ or absolute frequency of off-target mutations above which genome editors will be safe or below which they are not. One threshold that has been suggested is the background mutation rate in dividing cells, which has been estimated to be ~ 1.6 × 10^− 8^ [17]. This type of arbitrary threshold is irrelevant, however, because genome-editing activity is systematic and not random, so mutations at an off-target site that could inactivate a tumor-suppressor (such as P53) should be considered dangerous even at frequencies well below the background mutation rate, whereas high-frequency mutations in an inert non-coding region might be completely harmless.

Safety of therapeutic genome-editing approaches should be evaluated with a nuanced risk–benefit analysis. The obvious and greatest risk is unintended mutagenesis that confers cells with a proliferative advantage that leads to clonal expansion and malignant cellular transformation. In some cases, however, pro-proliferative mutations may serve to enhance the efficacy of treatment [18]. Some unintended effects may dampen the efficacy of a therapeutic strategy but may not be inherently dangerous. Pre-existing immunity to genome editors may result in the rapid clearance of edited cells, or an innate immune response to editing components could lead to cellular toxicity. The number and nature of cells that are exposed to genome editors is another risk modifier. Hundreds of millions of cells would typically be edited in ex vivo genome editing of human HSPCs or T cells, whereas in vivo editing of the liver could affect billions of cells. The greater the number of cells that are modified, the greater the possibility that one of them may accumulate undesired oncogenic driver mutations. Primary cells that have limited replicative potential may have a lower risk of transformation, whereas a deleterious mutation to a self-renewing stem cell may have long-term adverse consequences.

The potential benefits of genome-editing strategies may be more easily understood. A few notable examples include: 1) human T cells can be edited to disrupt *CCR5* and confer resistance to HIV infection [19]; 2) HSPCs from sickle cell disease patients can be modified to induce the expression of fetal hemoglobin as a functional replacement for defective adult hemoglobin in differentiated red blood cell progeny [2]; and 3) human T cells with enhanced tumor rejection properties can be engineered by targeted insertion of chimeric antigen receptors into the T-cell receptor alpha constant (TRAC) locus [1]. These benefits can be initially assessed through rigorous pre-clinical studies that measure the degree of efficient on-target editing and its functional consequences in cellular and animal models.

## State-of-the-art detection and prediction methods: Capabilities and limitations

Over the years, dramatic progress has been made in developing techniques to experimentally define the genome-wide activity of genome editors. These methods can be broadly divided into two categories: 1) cell-based strategies such as HTGTS (high-throughput, genome-wide translocation sequencing), BLESS/BLISS (breaks labeling, enrichment on streptavidin and sequencing/breaks labeling in situ and sequencing), GUIDE-seq (genome-wide unbiased identification of DSBs enabled by sequencing), and integrase-deficient lentivirus (IDLV) capture [20–25]); and 2) in vitro methods (CIRCLE-seq, Digenome-seq, and SITE-seq [26–29]), which we have previously reviewed in detail [15, 30]. The cell-based methods have the advantage of being able to detect cell-specific genome editing activity directly but have limitations in their sensitivity. In vitro methods are generally more sensitive and more comprehensive than cell-based methods, but characteristic nuclease-induced indel mutations cannot always be detected at all cleavage sites because of cell-specific chromatin accessibility, competition from endogenous cellular DNA–protein binding, or the concentration of genome-editing proteins that is achievable in cells.

Presently, the development of accurate and comprehensive computational or in silico methods for predicting genome-wide off-target activity is limited by the availability of large-scale training and validation datasets. Experimentally, off-target sites have been identified with up to six mismatches relative to their intended target site for CRISPR-Cas nucleases [20], up to eight mismatches for ZFNs [31], and up to 12 mismatches for TALENs [32]. Accurate in silico prediction of off-target activity is extremely difficult because the search space for potential off-targets is very large while the number of true off-targets is relatively small. Currently, it is possible to exclude particularly poor on-target sites that have closely related off-target sites by using computational tools such as Cas-OFFinder [33]. In the future, the generation of large-scale genome-wide genome-editing activity datasets, coupled with the development of machine-learning methods, may enable further progress in this challenging area. Until such in silico prediction methods mature and have been carefully vetted and prospectively validated, sensitive and unbiased experimental methods should be prioritized over in silico methods for defining the genome-wide activity of genome editors, because such experimental methods can sensitively and accurately identify sites without limiting pre-defined assumptions.

We should remain keenly aware of both the capabilities and limitations of the experimental methods that have been developed for discovering the genome-wide activity of genome editors. A common blind spot for both discovery and validation methods is their reliance on short-read high-throughput sequencing. Nearly 50% of the human genome is composed of repetitive elements [34], and so many regions remain difficult to uniquely map and are inaccessible to modern short-read, high-throughput sequencing methods [35]. Although they are difficult to sequence and map, repetitive elements are important as they often play an important role in tissue-specific gene regulation and host transcription-factor binding sites [36, 37]. Methods such as CIRCLE-seq that can identify full off-target sites in a sequencing read pair can overcome this mapping limitation, as they can be run in a reference genome-independent mode [26]. For validation, the error-rate of the high-throughput sequencing process, typically around 0.1%, can be limiting because it obscures mutational activity below this threshold. Two reports that found large deletions that were induced by CRISPR-Cas nucleases reinforce the point that our ability to detect genome-editing mutations is highly dependent on the method of observation [38, 39]. Using short-read, high-throughput sequencing technologies, large deletions, inversions, or structural rearrangements can easily be missed. Nuclease-induced DSBs can also interact with randomly occurring DSBs to generate chromosomal translocations [21].

Complementary methods should be used as required to obtain the broadest possible view of the activity of genome editors. When feasible in the cell types being studied, the pairing of cell-based methods such as GUIDE-seq with in vitro genome-wide activity profiling methods such as CIRCLE-seq or Digenome-seq may provide more information than either method alone. For validation of on-target and off-target activity, unidirectional anchored sequencing methods such as amplicon sequencing (AMP-seq) [40] and UDiTaS [41] may reveal information about structural rearrangements that cannot be observed using standard bidirectional PCR. Cytogenetic or other methods for visualizing large-scale genomic rearrangements may also play an important role in understanding the full impact of genome editing, revealing aspects that cannot be appreciated through the use of genomic sequencing methods alone. These methods may be especially important for genome-editing applications such as T cell-based cancer immunotherapy strategies where multiplex genome editing is often desirable (for example, to insert a chimeric antigen receptor and to knock out genes associated with T-cell exhaustion simultaneously).

Cell-based surrogate assays (where the cells used to analyze specificity do not match the target cell type) should be avoided because they do not account for genetic or epigenetic differences between the surrogate and the target cell type. There may be differences in epigenetic factors or chromatin organization between the surrogate and target cells. In certain challenging primary cell types such as human hematopoietic stem cells (HSCs), where it is difficult to use assays such as GUIDE-seq, a combination of in vitro discovery and targeted validation is preferable.

Genome-wide assays to define genome-editing activity should be designed to read out the enzymatic activity of interest as directly as possible. In widely used *Streptococcus pyogenes* Cas9, DNA cleavage is allosterically regulated by extensive RNA–DNA complementarity beyond that required for binding [42, 43]. Therefore, assays such as chromatin immunoprecipitation sequencing (ChIP-seq) that can be used to measure the binding of catalytically inactive or dead Cas9 (dCas9) are not generally predictive of genuine Cas9 cleavage sites [42]. Similarly, nuclease-induced mutagenesis is not necessarily correlated with base editing, which depends largely on the DNA-binding and helicase activities of Cas9 [44]. These examples illustrate why assays that are designed to read out the catalytic or mutational activity of the genome editors themselves are crucial and likely to be more informative than other studies.

An eyes-wide-open approach to defining the fundamental genome-wide activity of genome editors should inspire not diminish confidence in their safety*.* Increased assay sensitivity does not imply that all genome editors are flawed but should be considered as the means for rational data-driven selection of editors that are truly the best choice for each clinical application. For example, these highly sensitive state-of-the-art methods enable rigorous examination of the relative merits of engineered variants or newly discovered genome editors.

## A framework for predicting functional mutation sites

**Fig. 1:**
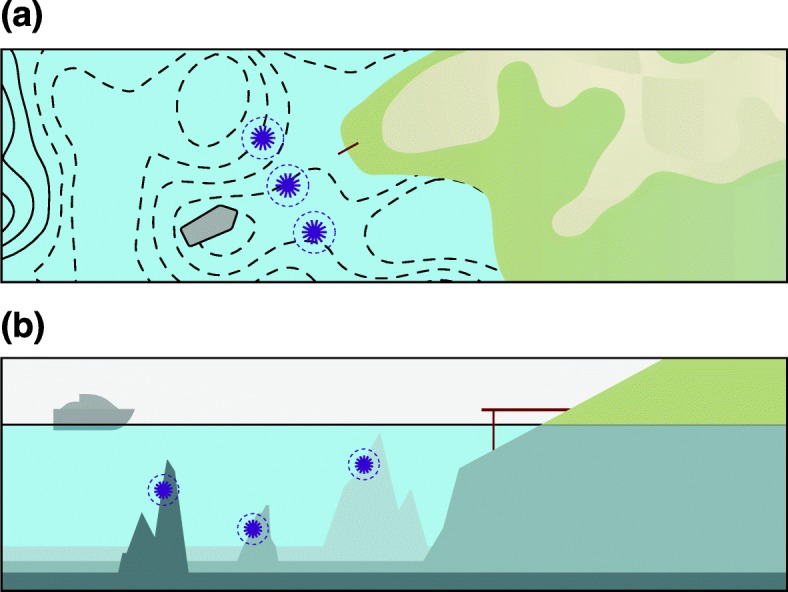
Charting a course towards safe genome editing. **a** Like an incomplete nautical map, current methods for defining the genome-wide activities of genome editors identify the locations of potential hazards without offering additional insight into the level of functional risk. **b** Methods that look below the surface will allow us to understand the level of risk associated with particular hazards and will help to increase confidence in genome-editing strategies

Risks associated with genome editing should be considered as an integrated measure of the location, frequency, and functional impact of the resulting off-target activity. Significant advances have been made through the development of sophisticated genome-wide methods to determine the location and frequency of unintended off-target activity, but discerning functional impact remains a major challenge. Our understanding of the genome-wide activity of genome editors remains superficial, like an incomplete nautical map showing potential hazards without indicating how dangerous they might be. To chart a safe course towards genome-editing therapeutics, it will be important to develop new methods that will allow us to see below the surface and functionally understand the consequences of genome-editing activity (see Fig. 1). The question is: how can we distinguish harmful from benign sites of off-target mutations? Here, we discuss how off-target mutations may affect normal genome functions and propose criteria for the design of therapeutic genome editing.

Obviously, off-target sites that are located within protein-coding sequences are most likely to have a functional impact and should be avoided if possible. Small indels are the most common type of mutations introduced by genome-editing nucleases, and these mutations can lead to the frameshift of protein-coding sequences and functional gene knockout. Indels that are close to the 5′ end of transcripts start sites or within functional domains would be predicted to cause more severe side effects. Although avoidance of off-target mutations within protein-coding sequences is preferred, there may be exceptions to this rule. Not all genes are actively expressed in a given cell type, so genome editors that induce off-target mutations that are located within silenced genes may still be considered, especially if no better alternatives are available. In some cases, off-target mutations in protein-coding sequences that are closely related to the target sequence may be unavoidable but acceptable because they are benign. As protein-coding sequences contribute to less than 2% of the human genome, we anticipate the vast majority of off-target mutations will be found in non-coding DNA sequences. To date, our knowledge of the function and organization of non-coding sequences remains elusive, further increasing the difficulty of accurately predicting the functional consequences of mutations at non-coding off-target sites.

**Fig. 2:**
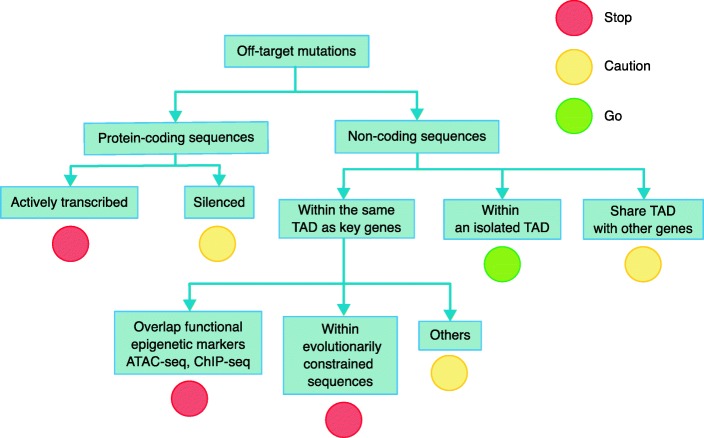
Functional classification of sites of off-target genome-editing mutations. A proposed framework outlining principles for defining the function of sites of off-target mutation. Some sites should be strictly avoided (red), others may require caution in interpretation (yellow), and a few in isolated topologically associated domains (TADs) may be non-functional and unlikely to be deleterious (green)

Although there is still no gold standard for categorizing deleterious non-coding mutations, we can outline some foundational principles for assessing off-target activity in these regions (see Fig. 2). First, epigenetic signals such as histone modifications (H3K27ac, H3K4me1, and H3K4me3), chromatin openness, and transcription factor occupancy have been widely used as markers for active regulatory DNA sequences [45–47], and genome editors that induce off-target mutations overlapping these features should be avoided. Second, DNA sequences that are under strong purifying selection or positive selection are likely to be associated with important biological functions and should not be modified either. Evolutionarily constrained regions of the human genome are highly enriched in pathogenic variants and new maps of these sequence constraints from thousands of people may help to infer the locations of important non-coding genetic elements [48]. Third, because the functions of non-coding sequences are highly tissue- or cell type-specific, the evaluation of non-coding mutation effects should be conducted in the context of the edited cell type. The human genome is spatially organized into different units called topologically associating domains (TADs). Most interactions between regulatory sequences and target genes occur within the same TAD [49, 50]. Thus, the prediction of non-coding mutations needs to be conducted in the context of TAD structure.

Nevertheless, an approach of avoiding all genomic loci that overlap with DNA sequences enriched with epigenetic signals may be overly cautious as there is no guarantee that all such sequences will have regulatory functions. Although the impact of indels on coding sequences could be highly disruptive, small indels may not be sufficient to disrupt the functions of many non-coding regulatory sequences [51] and the natural occurrence of polymorphisms within those loci among the healthy population might suggest that a considerable percentage of those mutations are actually functionally neutral [52].

## Present and future outlook for genome-editing therapeutics

Some published reports have been interpreted as indicating a need for concern about the prospects of certain therapeutic genome-editing technologies. Schaefer et al. [53] initially claimed that Cas9 induces genome-wide point mutations and two groups reported that CRISPR-Cas9-mediated DSBs activate a TP53 response that had to be suppressed before they could achieve efficient genome editing in certain cell types [54, 55]. In our view, therapeutic genome editing should continue to be approached rigorously and carefully, but there is no overt cause for alarm.

The Schaefer et al. [53] report, which has subsequently been retracted, claimed that CRISPR-Cas nucleases induce high-frequency point mutations genome-wide. Instead, because the genetic relationship between the edited and control mice remains unclear, the simplest explanation for the genetic differences observed is pre-existing heterogeneity in the genetic background of the mice that were involved in this study [56–60]. Careful follow-up studies employing trio sequencing of genome-edited mice found no evidence of unexpected Cas9-induced point mutations at levels above background [61].

Most currently envisioned clinical genome-editing strategies do not depend on TP53 inhibition or genetic selection of modified cells. In many cases, clinical strategies plan to edit large numbers of primary cells such as HSPCs or T cells ex vivo for later direct reinfusion into the patient. As genetic selection for correctly modified cells is typically not feasible and not performed, there is no increased risk of enriching for cells that have previously acquired TP53 mutations. Nevertheless, there is an exception in situations where the edited cells have a strong selective advantage over unedited cells. An example of this is the gene correction of *IL2RG* for X-linked severe combined immunodeficiency (SCID-X1), where *IL2RG-*corrected B and T cells have a strong advantage over *IL2RG* mutant cells in repopulating the thymus. In these special cases, it may be important to achieve high editing efficiency in a number of cells that is sufficient to minimize the possibility of selectively expanding cell clones harboring unwanted tumorigenic mutations [62–64].

For clinical genome editing, it may be important to account for genetic variation between individuals, but the impact of this variation will need to be ascertained experimentally. Certainly, all practitioners will take into account and typically avoid on-target sites in which there is common genetic variation. There are clear reports of sites at which individual single nucleotide variants can affect the activity of genome editors [26, 65], but the general impact of human genetic variation on genome-wide activity is less clear. Understanding these effects will require the development of scalable, high-throughput versions of sensitive and unbiased genome-scale methods to define the genome-wide activity of genome editors. With better tools, we anticipate that it may become routine to check the genome-wide activity of editors in the context of an individual’s specific genomic DNA.

Over the past several years, remarkable progress has been made not only in fundamental genome-editing technologies but also in the tools used to illuminate their genome-wide editing activity. These methods serve the important purpose of highlighting locations of unintended mutagenesis and have enabled the careful selection of clinical genome-editing strategies and targets that are now progressing through human clinical trials. Although we can now see the unintended mutagenic activity of genome editors in living cell genomes much more clearly, an important future challenge will be to develop new ways to interpret the functional biological consequences of this activity. Advances in our capability to illuminate and interpret global genome-editing activity will inspire confidence in the safety of the next generation of promising genome-editing therapies.

## Abbreviations

CRISPR: Clustered regularly interspaced palindromic repeats
DSB: Double-strand break
HSPC: Hematopoietic stem and progenitor cell
indel: Insertion/deletion
TAD: Topologically associated domain
TALEN: Transcription activator effector-like nuclease
ZFN: Zinc-finger nuclease
